# Evaluation of nitazoxanide treatment following triclabendazole failure in an outbreak of human fascioliasis in Upper Egypt

**DOI:** 10.1371/journal.pntd.0007779

**Published:** 2019-09-25

**Authors:** Haidi Karam-Allah Ramadan, Waleed Attia Hassan, Nahed Ahmed Elossily, Alzahraa Abdelraouf Ahmad, Adnan Ahmed Mohamed, Alaa Soliman Abd- Elkader, Eman M. Nagiub Abdelsalam, Hani M. J. Khojah

**Affiliations:** 1 Department of Tropical medicine and Gastroenterology, Faculty of Medicine, Assiut University, Egypt; 2 Department of Parasitology, Faculty of Medicine, Assiut University, Egypt; 3 Department of Clinical Pathology, Faculty of Medicine, Assiut University, Egypt; 4 Department of Clinical and Hospital Pharmacy, College of Pharmacy, Taibah University, Madinah, Saudi Arabia; Queen’s University Belfast, UNITED KINGDOM

## Abstract

**Background:**

Fascioliasis is a neglected zoonosis with major public health implications in humans. Although triclabendazole (TCBZ) is the drug of choice, there are records of TCBZ failure worldwide. TCBZ-resistant fascioliasis is treated with alternative approved drugs including nitazoxanide (NTZ), with varying levels of efficacy. Data on NTZ efficacy after TCBZ failure in Egypt is scarce. This study evaluated the efficacy of NTZ in cases of TCBZ failure during an outbreak of fascioliasis in Assiut governorate of Upper Egypt.

**Methodology/Principal findings:**

This prospective study included 67 patients from the outpatient clinic in Manfalout locality of Assiut governorate with clinical manifestations of acute fascioliasis. These included high eosinophilia (> 6% eosinophils in peripheral blood), positive anti-*Fasciola* antibodies, and hepatic focal lesions (HFL) or ascites on abdominal ultrasound or computed tomography. All patients initially received TCBZ at recommended doses. Patients were followed up after 1 month to assess response. According to the responses, patients were categorized as non-responders and responders. The non-responders received a trial of NTZ and were re-assessed for response based on clinical manifestations, eosinophil count, and abdominal ultrasound. Patients not responding to NTZ received additional doses of TCBZ.

One month after initial TCBZ treatment, 37 patients responded well to TCBZ, while 30 patients failed to respond with persistence of fever, abdominal pain, high eosinophilia, and HFL. Most non-responders were male (56.7%); females predominated among TCBZ responders (62.2%). The mean age of the non-responders was relatively lower, at 20.57 ± 14.47 years (p = 0.004). Following NTZ therapy, HFL disappeared in 9/30 (30%) patients and eosinophil counts normalized in only 2 (6.7%) patients, indicating an overall efficacy of 36.6%. The remaining cases received additional doses of TCBZ with complete clinical, pathological, and radiological resolution.

**Conclusions/Significance:**

Nitazoxanide was partially effective in TCBZ failure in acute human fascioliasis in Upper Egypt. Further studies with larger samples are highly encouraged and further research is urgently needed to find new therapeutic alternatives to TCBZ.

## Author summary

Fascioliasis is a neglected zoonosis with major public health implications in humans. Triclabendazole (TCBZ) is the drug of choice, but alternative approved drugs are necessary in cases of TCBZ failure. Nitazoxanide (NTZ) is an alternative used in such cases. However, the efficacy of NTZ in TCBZ-failure cases among patients in Egypt remains unclear. In this study, the efficacy of NTZ was evaluated in cases of TCBZ failure during an outbreak of human fascioliasis in Assiut governorate of Upper Egypt. This study enrolled 67 patients diagnosed with fascioliasis based on clinical, laboratory, and radiological findings. These patients were referred from the outpatient clinic in Manfalout locality of Assiut governorate in Egypt. All patients received TCBZ at recommended doses as initial treatment. Those failing to respond were treated with NTZ at standard doses; following therapy, lesions in the liver and high eosinophil counts were resolved in 30% and 6.7% patients, respectively, indicating an overall efficacy of 36.6%. Therefore, in this outbreak of human fascioliasis in Upper Egypt, NTZ was found to be partially effective in cases with TCBZ failure.

## Introduction

Fascioliasis has emerged as a notable zoonotic disease with considerable impact on veterinary and public health. This prompted the World Health Organization (WHO) to include human fascioliasis among the important neglected tropical diseases (NTDs) [1]. It is a foodborne disease caused by trematodes belonging to the genus *Fasciola* (*F*. *hepatica* and *F*. *gigantica*). In the past few decades, the incidence of human fascioliasis has considerably risen in different parts of the world. The rise has been particularly remarkable in South America, Asia, and Africa including Egypt, where the two common species of *Fasciola* coexist [2]. Recent studies have revealed a large number of cases of fascioliasis (2.4 to 17 million cases) worldwide [3].

Fasciolids are parasites of the hepato-biliary ducts, and the disease is mostly confined to the liver. Therefore, the main pathogenic sequelae are hepatic lesions, fibrosis, and chronic inflammation of the biliary passages. The pre-patent period together with the time to onset of signs/symptoms of the disease may range from a few days to 2–3 months or longer. There are 2 main clinical stages in fascioliasis. The acute stage coincides with larval migration and mechanical destruction of the liver tissue. This stage extends till worm maturation in the hepatic tissues, and lasts for 2–4 months. The chronic stage coincides with the persistence of adult *Fasciola* worms in the bile ducts and may last for months or even years [4]. Eosinophilia is the most common clinico-pathological feature against fascioliasis in both stages.

In Egypt, fascioliasis has probably been prevalent for a very long period, since the times of the pharaohs [5, 6]. High levels of infestation have been widely described in livestock, [7] resulting in considerable economic losses and expenditure for purchase of anthelmintics, liver condemnation, loss of production due to mortality, lower production of meat, milk, and wool, reduced weight gain, and impaired fertility [8].

The mainstay of treatment in fascioliasis affecting animals and humans is triclabendazole (TCBZ), which targets both the immature stages and mature adult worms [9]. Older drugs, such as tetrachloride, tetrachlorethylene, and bithionol, are currently considered to be less effective, unacceptably toxic, or both [10]. Although TCBZ is the only effective treatment for fascioliasis, it is currently registered for human use in only 4 countries [11].

The widespread use of TCBZ in the livestock industry led to the emergence of resistance in fluke populations affecting ruminants in both, developed and developing countries including Ireland, Spain, Australia, Peru, and Argentina [10]. The zoonotic nature of fascioliasis may raise concerns regarding the transmission of resistant strains to humans, particularly in endemic areas such as Peru, Bolivia, and Egypt [12]. In recent years, a few reports have described the occurrence of TCBZ resistance in humans. The first case was reported in a livestock farmer in the Netherlands, followed by 4, 1, and 7 cases in Chile, Turkey, and Peru, respectively [13–16]. Unfortunately, despite the prevalence of TCBZ resistance in Egypt, a review of the literature does not reveal any published data.

Reliance on monotherapy poses a risk for the treatment of fascioliasis, particularly in the absence of a vaccine for the prevention of the disease [9]. As cases of TCBZ resistance are continuously being documented from livestock, human cases of TCBZ-resistant fascioliasis are most likely to occur. This is a serious challenge for treatment in humans, with considerable public health implications [8] and emphasizes the urgent need for developing new fasciocidal drugs [17].

Several trials were conducted in the search for effective alternative drugs for fascioliasis. Nitazoxanide (NTZ), which is a broad spectrum antiparasitic agent, has been found to be well tolerated by humans, with adverse effects similar to that of placebo [10]. Across different studies, its efficacy has ranged from 40–100% [18].

The aim of the present study was to investigate the efficacy of nitazoxanide as a treatment for fascioliasis in the face of incomplete response to triclabendazole in Upper Egypt.

## Methods

### Study design

This prospective study was conducted between August and November 2018.

### Ethical approval and consent

The study protocol was approved by the Institutional Review Board of the Faculty of Medicine of Assiut University, Egypt. Written informed consent was obtained from all patients prior to participation in this study.

### Study population

A total of 74 patients with diagnosed or suspected fascioliasis were recruited in the study. All these patients were referred to the outpatient clinic in the Tropical Medicine and Gastroenterology Department at the Al-Rajhi Liver University Hospital during an outbreak of fascioliasis in Manfalout locality of Assiut Governorate in Upper Egypt.

### Clinical and laboratory investigations

The included patients had symptoms and signs suggestive of fascioliasis such as fever, abdominal pain, jaundice, and hepatomegaly. The complete blood count (CBC), including eosinophil percentage and absolute eosinophil count was individually assessed using the ADVIA 2120i Hematology System (Siemens Healthcare Diagnostics Inc. Tarrytown, NY 10591, USA). Stool examination was also performed for all cases for the qualitative diagnosis of fascioliasis using the native lugol and formalin ethyl acetate sedimentation method [19]. Stool samples were also examined for the presence of other co-existing intestinal parasites that could potentially cross-react or overlap with fascioliasis. Liver function tests were also performed.

Further investigations included; serological analysis was done by *F*. *hepatica* IgG Enzyme-linked immune sorbent assay (ELISA) kits (DiaColon Tech Houston, USA) for qualitative diagnosis of fascioliasis. The result was read photometrically at 450 nm (TECAN Sunrise Absorbance Reader). (values greater than 10.0 AU/ml were interpreted as seropositive, cut-off value 0.25 according to the manufacturer’s instructions) Indirect hemagglutination assay (IHA) using Distomatose Fumouze (Laboratories Fumouze Diagnostic, Levallois Perret, France) was also done to compare antibody titers (a titer ≥ 1/320 was considered to be positive). Abdominal ultrasound (US), and abdominal computerized tomography (CT) were also done. Endoscopic retrograde cholangiopancreatography (ERCP) was performed in cases presenting with obstructive jaundice and a dilated common bile duct (CBD) on abdominal US and/or CT.

### Treatment regimen and follow up of patients

All patients received a double dose of triclabendazole (Egaten, Novartis Pharma AG) at a dose of 10 mg/Kg/dose, at 12-hour interval in a joint venture with the WHO. Patients were advised to avoid vegetables that posed a risk for re-infection.

The endpoints for treatment response were evaluated on follow up after 1 month. Evaluation was based on 3 parameters, namely, resolution of clinical symptoms and signs, normalization of eosinophil counts, and improvement of hepatic lesions on US. According to the WHO criteria, persistence of symptoms or signs with either eosinophilia (> 6% eosinophils in peripheral blood) or hepatic focal lesions, was considered to be a probable indicator of treatment failure with TCBZ [1]. Patients were then divided into 2 groups according to treatment response. The first group included the patients who did not respond to TCBZ and were administered NTZ (non-responders), while the second group included patients who successfully responded to TCBZ (responders).

The non-responders received NTZ at a dose of 500 mg orally every 12 hours for 7 days. Patients were clinically assessed for response after 1 month. Resolution of both, eosinophilia in the CBC and/or hepatic focal lesions on US were indicative of response. Patients who failed to respond to NTZ were re-treated with TCBZ at doses similar to the initial dose and were followed up for response.

Patients who received any other anthelminthic drugs within 1 month before TCBZ or NTZ therapy including bithionol, praziquantel, albendazole, dihydroemetine, or emetine hydrochloride, and patients who showed hypersensitivity to nitazoxanide were excluded from this study.

### Statistical analysis

Data entry and analysis were performed using the IBM SPSS Statistics for Windows, Version 20.0. (Armonk, NY: IBM Corp) software package. Data were presented as numbers, percentages, means, and standard deviations. The Chi-square and Fisher’s exact tests were used to compare qualitative variables. The Mann-Whitney test and the Wilcoxon signed rank test were used to compare variables between independent and dependent groups, respectively. In case of non-parametric data, the Wilcoxon signed rank test was used to compare the quantitative variables before and after treatment. A P-value < 0.05 was considered statistically significant.

## Results

In this prospective study, 74 patients were initially recruited. Among them, 67 patients with symptoms and signs suggestive of acute fascioliasis were included for the NTZ trial; 7 patients were excluded as they presented with obstructive jaundice and a dilated CBD on ultrasound (suggesting chronic fascioliasis). These 7 patients underwent endoscopic sphincterotomy and extraction of the adult worm by ERCP followed by TCBZ therapy. The included patients received initial treatment with a double-dose of TCBZ.

**Fig 1:**
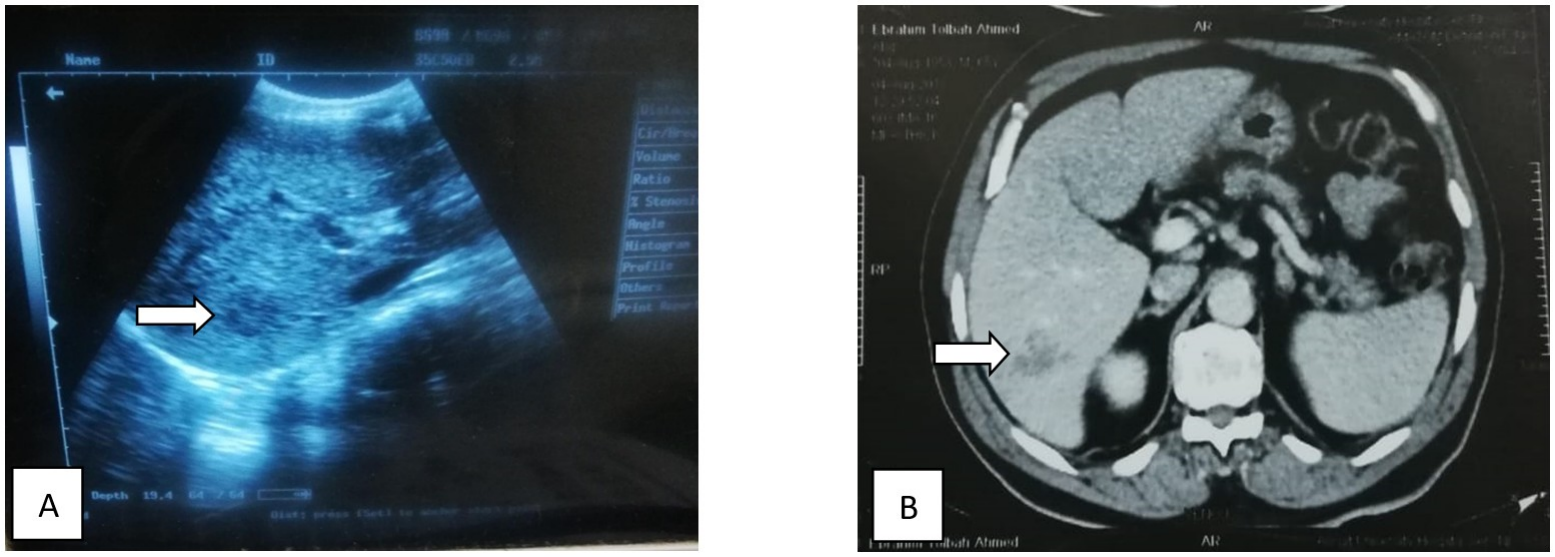
A. Ultrasound image; B. Abdominal CT image of a hepatic focal lesion (HFL) caused by fascioliasis in the right lobe (white arrows).

The pretreatment demographic, clinical, and laboratory data of the studied patients are shown in Tables 1–3, respectively. The cohort comprised 31 male and 36 female patients with a mean age of 26.27±15.3 (range: 4–60) years. The patients presented with one or more of the symptoms and signs of acute infection, which include fever, abdominal pain, hepatomegaly, splenomegaly, and ascites. Laboratory data showed mild anemia (hemoglobin [Hb]: 11.8± 0.7 g/dl), high eosinophilia (41.1 ± 15.7%), high alanine transaminase (ALT) and aspartate transaminase (AST) levels, and a positive serological titer (936.1±387.2). As depicted in Fig 1, radiological investigations showed the presence of hepatic focal lesions (HFL) in 25 patients (37.3%). Stool examination was positive for *Fasciola* eggs in 7 of 67 patients (10.4%) with absence of other co-existing parasitic infections that could, potentially, construct immunological cross-reactions or clinical symptoms overlapping with fascioliasis.

**Table 1:** Pretreatment demographic data of the studied patients.

	Number	Percentage (%)
Age		
4–17 years	22	32.8
18–40	32	47.8
41–60	13	19.4
Sex		
Male	31	46.27
Female	36	53.73

**Table 2:** Pretreatment clinical data of the studied patients.

	Number	Percentage (%)
Fever	50	74.3
Abdominal pain	66	98.5
Hepatomegaly	26	38.8
HFL	25	37.3
Splenomegaly	11	16.4
Ascites	12	17.9

**Table 3:** Pretreatment laboratory data of the studied patients.

	Number	Percentage (%)
Anemia	37 (Hb = 11.1 ± 0.7 g/dl)	55.2
High Eosinophilia	67	100
Range	(41.1 ± 15.7%)	
High ALT	32	47.8
Range	(67.4 IU/L ± 36.1)	
High AST	28	41.8
Range	(67.8 IU/L ± 28.9)	
Positive egg in stool	7	10.4

Hb: Hemoglobin, ALT: Alanine aminotransferease; AST: Aspartate aminotransferase; HFL: Hepatic focal lesions. Reference ranges for Hemoglobin are in Male: 14–17 g/dL Female: 12–16 g/dL, for eosinophilia 0–5%, for ALT (0–35 IU/L) and for AST (0–35 IU/L).

**Fig 2:**
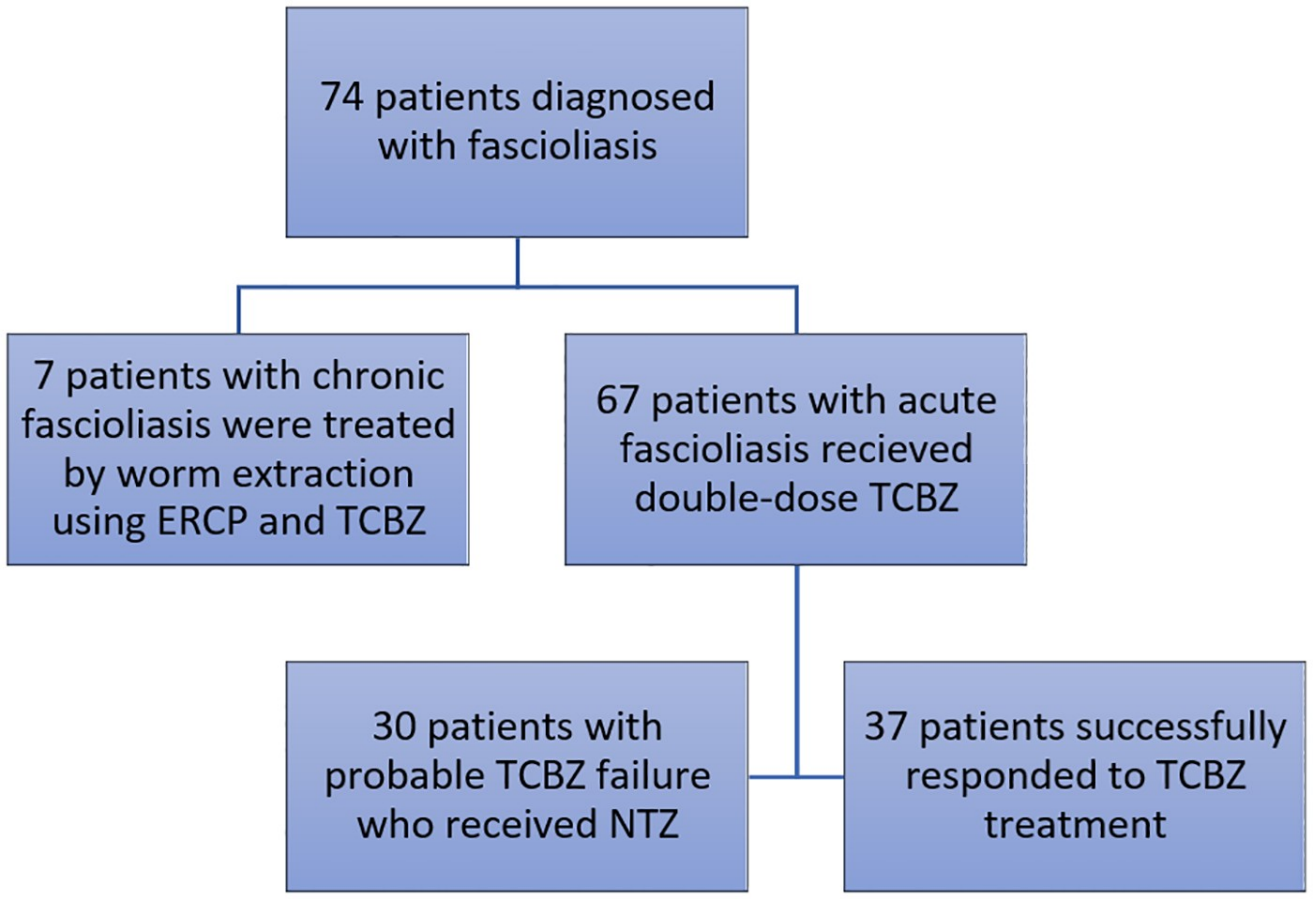
The flow chart of treatment in the patients included in the study.

The studied patients were followed up after 1 month to evaluate the response to first line TCBZ. A total of 37 cases (55.2%) showed good response to TCBZ (the responder group) as evidenced by disappearance of signs and symptoms, normalization of peripheral eosinophil counts, and resolution of HFL. The remaining 30 cases (44.8%) (the non-responder group) showed persistence of infection, as evidenced by persistence of clinical manifestations, high eosinophilia, and HFL. This group received nitazoxanide and were followed up after 1 month (Fig 2).

The demographic, clinical, and sonographic characteristics of both groups, as summarized in Table 4, showed that most patients in non-responder group were male (56.7%), while females were predominant in the responder group (62.2%). Also, the mean age in the responder group (30.89 ± 14.57 years) was significantly higher than that of the non-responder group (20.57 ± 14.47 years) (p = 0.004). However, the clinical presentation and sonographic evidence of HFL were not significantly different between the groups.

**Table 4:** Demographic, clinical, and sonographic characteristics of patients who received first line triclabendazole.

		Non-responders to TCBZ (n = 30)		Responders to TCBZ (n = 37)		P value
		No.	%	No.	%	
**Sex**:	**Male**	17	56.7	14	37.8	0.124
	**Female**	13	43.3	23	62.2	
**Age (years)**	**Mean**± **SD**	20.57 ± 14.47		30.89 ± 14.57		0.004*
	**Range**	4–60		6–56		
**Clinical data**	**Fever**	25	83.3	25	67.6	0.140
	**Abdominal pain**	29	96.7	37	100.0	0.448
	**Hepatomegaly**	13	43.3	13	35.1	0.493
	**Splenomegaly**	5	16.7	6	16.2	1.000
	**Ascites**	8	26.7	4	10.8	0.092
**Sonographic data**	**HFL**	14	46.7	11	29.7	0.154

* Significant *p*-value ≤ 0.05

As shown in Table 5, the hematological, biochemical, serological and parasitological parameters of patients at baseline were not considerably different between patients in both groups, except for total leucocyte count, and levels of ALT and AST, that were significantly higher in the non-responder group (p = 0.008, p = 0.026, and 0.047, respectively).

**Table 5:** Laboratory data of the studied patients who received first line triclabendazole.

	Non-responders to TCBZ (n = 30)	Responders to TCBZ (n = 37)	*P* value
	Mean ± SD	Mean ± SD	
**Hb level** (g/dl)	11.80 ± 1.13	11.81 ± 0.90	0.930
**WBCs** (cells/μL)	17.46 ± 12.25	10.27 ± 3.92	0.008*
**Eosinophil % before treatment Range**	26.72 ±13.21 14–40%	30.47±15.18 7–70%	0.081
**Eosinophil % after treatment Range**	20.00±11.28 3–59%	3.6±1.7 1–15%	0.0001*
**PLT** (cells/μL)	311.60 ± 85.07	308.11 ± 75.79	0.796
**Bilirubin** (mg/dl)	1.04 ± 0.15	1.06 ± 0.17	0.613
**ALT** (IU/L)	61.43 ± 43.44	39.62 ± 18.52	0.026*
**AST** (IU/L)	53.83 ± 36.05	35.78 ± 15.84	0.047*
**Positive stool analysis**	2 (6.7%)	5 (13.5%)	0.447
**Serology titer**	885.33 ± 391.32	977.30 ± 384.19	0.338

Hb level: hemoglobin level; WBCS: White blood cells; PLT: platelets count; ALT: Alanine aminotransferease, AST: Aspartate aminotransferase. Reference ranges for Hemoglobin Male: 14–17 g/dL Female: 12–16 g/dL, WBCs 4000–10,000/μL, for eosinophilia 0–5%, Platelet count 150,000–350,000/μL, for Total bilirubin—0.3–1.2 mg/dL, for ALT (0–35) IU/L, for AST (0–35) IU/L. Serology titer ≥ 1/320 is considered positive

* Significant *p*-value by Fisher exact test.

Furthermore, the assessment of response to first line TCBZ showed complete resolution of the clinical manifestations in all patients in the responder group; patients in the non-responder group had persistent fever and abdominal pain. The pre-treatment eosinophil counts were not significantly different between the groups (p = 0.081). After treatment, limited improvement in eosinophil counts was observed in the non-responder group, with a reduction from 26.72% ± 13.21 to 20.00% ± 11.28. In the responder group, the counts reduced from 30.47% ± 15.18 to 3.6% ±1.7, showing statistically significant difference between the groups (p = 0.000).

After NTZ treatment in the non-responder group, HFL disappeared in 9/30 patients (30%) as opposed to all patients in the TCBZ responder group; this difference was statistically significant (p = 0.015) (Table 6). In addition, eosinophil counts normalized in only 2 (6.7%) patients after NTZ therapy. Patients who did not show improvement after NTZ therapy received an additional dose of TCBZ, similar to the initial dose, with complete clinical, laboratory, and radiological resolution. Therefore, based on the improvement of eosinophil counts and HFLs in patients with TCBZ failure, nitazoxanide was effective in 11/30 patients (36.6%).

**Table 6:** Comparison of improvement in hepatic focal lesions (HFLs) in patients before and after treatment with triclabendazole and nitazoxanide.

HFLs	Patients who received NTZ (n = 30)		Patients who responded to TCBZ (n = 37)		P-value
	No.	%	No.	%	
**Before treatment**	14	46.7	11	29.7	0.154
**After treatment**	9	36.6	0	0.0	0.015*

* Significant *p*-value by Fisher exact test.

## Discussion

Owing to its activity against juvenile and adult forms of the parasite, TCBZ is the drug of choice in the treatment of *F*. *hepatica* and *F*. *gigantica* infections in humans [1]. Mass control programs for human fascioliasis in Egypt, Vietnam, Bolivia, and Peru have used TCBZ, which was donated through an agreement between the WHO and the manufacturer [20].

Several previous studies have documented the clinical efficacy of TCBZ with various treatment regimens in different regions including Egypt [21–23]. The results of these drug trials are indicative of a dose–response relationship. The WHO currently recommends the administration of a single dose of TCBZ at a dose of 10 mg/kg for the treatment of human fascioliasis, and a double dose of 10 mg/kg, 12 hours apart, in severe cases [1]. In a randomized open-labeled study conducted in Egypt, which compared 1- and 2- dose regimens of TCBZ at 10 mg/kg, the 2-dose regimen showed more favorable results [21].

Indeed, TCBZ is the only first-line medication with reports of high efficacy in humans. Therefore, the effective management of resistance to this drug is of utmost importance [9]. Clinical trials on alternatives to TCBZ are limited. This is probably the first study to evaluate the efficacy of NTZ in the management of cases of acute fascioliasis with TCBZ failure in Egypt.

In the current study, all cases of acute fascioliasis were defined based on clinical manifestations, high eosinophilia, and radiological signs with positive anti-*Fasciola* antibodies. However, stool examination was positive in only 7 cases (10.4%) with a low egg burden. In the present study we could not rely only on coprological examination for the diagnosis and follow up of cases. This is attributed to many factors, including prepatent or acute infections (where the patients were symptomatic prior to the appearance of eggs in the stool) [24], the inability of adult *Fasciola* worms to produce eggs (due to its lack of adaptation to the human host), encapsulation of eggs in granulomas or abscesses in the liver, and low egg shedding related to low infection burdens [25]. Coprological examination may also overestimate the response to treatment since the age of the fluke or its anatomic location, which may be associated with increased susceptibility to treatment, may impact the results [26].

In a study previously conducted in Egypt including 23 cases, *Fasciola* eggs were detected in only 2 cases (8.6%) as the patients were diagnosed in the hepatic phase [27]. An immature worm feeds on liver tissue without producing eggs; the only evidences of infection are eosinophilia and HFL, which are observed in early stages of the infection [28].

The detection of anti-*Fasciola* antibodies by the ELISA test is a reliable and sensitive test for diagnosis of fascioliasis compared with stool examination. The main advantage is that results are positive as early as 2 weeks post infection. However, since serum antibodies may persist for 4–5 months after successful treatment, it is not a reliable test in the evaluation of response during follow up [24]. Eosinophilia as a host defense mechanism is a common feature of fascioliasis and is encountered in 14%- 82% of patients, and may rise and fall during the chronic stage [29].

As described by Marcos et al., the primary outcome measures for clinical cure after treatment are defined by resolution of the clinical picture and eosinophilia during follow up [30]. Therefore, in the current study, post-treatment follow-up was based mainly on the persistence of clinical manifestations with either high eosinophilia with or without radiological signs.

In the present study, as evidenced by the disappearance of signs and symptoms, normalization of peripheral eosinophil counts, and resolution of HFL, 37 patients (55.2%) showed good response to TCBZ. The remaining 30 cases (44.8%) were suspected to have TCBZ failure and were treated with NTZ. The mean age of the non-responder group was lower than that of the TCBZ responders; this may have had an impact on the treatment response. A double blinded placebo-controlled trial in Peru, which employed NTZ for the treatment of chronic fascioliasis, has shown a low cure rate in children (40%) and a slightly higher efficacy in adults (60%) [9].

In our cohort, most non-responders were male (56.7%); females were predominant among the responders (62.2%). The gender of the studied patients did not significantly differ between groups. However, previous studies have indicated differences in sensitivity to flukicides depending on the sex of the host animals infected with *F*. *hepatica* [31].

Notably, in the current study, patients who did not initially respond to TCBZ in the acute stage, responded to the subsequent trial of TCBZ administered 2 months after the initial dose, in the chronic stage. This relationship between response to TCBZ and the stage of the disease has also been previously mentioned by Marcos et al. [30], who reported the amelioration of eosinophil counts after a single dose of TCBZ in 10 patients with acute *Fasciola* infection. However, parasitological cure (the absence of eggs in the stools) was not reported during follow up.

The difference in TCBZ susceptibility between juvenile and adult parasites has been previously described in an in vitro study with *Fasciola hepatica* infection [17]. However, this has not been thoroughly described in case series including patients with acute fascioliasis [32, 33].

According to our results, 30 patients showed clinical evidence of the presence of TCBZ-resistant *F*. *hepatica* infection, which is considered a large number. They received a trial with NTZ at a dose of 500 mg twice daily for 7 days, that showed an overall efficacy of 36.6% (11/30 patients), based on the improvement of eosinophil counts and HFLs.

NTZ has been widely used in the management of different parasitic infections with reportedly high efficacy and tolerability. The efficacy of NTZ against *Fasciola* has been studied in rabbits experimentally infected with *F*. *gigantica*. NTZ was found to be partially effective (47%) against the juvenile stages of the parasite, but completely effective (100%) against the adult stage [34].

A few clinical trials have been conducted on the efficacy of NTZ in the treatment of human fascioliasis with considerably variable efficacy. In Egypt, an open-label clinical study including 125 Egyptian patients with chronic fascioliasis demonstrated 97% clearance of *F*. *hepatica* eggs in the stool on day 30 after treatment with NTZ; the serological and eosinophilic patterns had also improved [35]. A second report from Egypt showed a slightly lower cure rate with NTZ (82.4%) [36]. Similar results were observed in a study conducted on schoolchildren in Mexico that documented the efficacy rates of NTZ against chronic fascioliasis to be 94.0% and 100% after the first and second treatment courses, respectively [37].

A much lower efficacy rate was observed in a double-blinded placebo-controlled study in northern Peru, where 50 adults and 50 children infected with *F*. *hepatica* received a 7-day course of NTZ. Compared to the placebo group, 60% adults and 40% children were cured [10]. These results suggest that NTZ may be a reasonable option at least in the chronic stage of fascioliasis, and is a good alternative to TCBZ.

Conversely, some studies have revealed a lack of efficacy of NTZ in 24 cases of liver fluke infection in Cuba [38] and in a patient with apparent TCBZ failure in the Netherlands [13]. Cabada and colleagues have reported that a cohort of 7 patients, infected by ingesting watercress in the Cusco region of Peru, had failed to respond to multiple courses of TCBZ in combination with NTZ [16]. The wide variances in fasciolid susceptibility to NTZ may be attributed to differences in geographical strains of *Fasciola* in various regions [16]. This indicates the urgent need for further controlled clinical trials to evaluate the efficacy of NTZ in the control of fascioliasis.

Although TCBZ resistant fascioliasis has been widely described in livestock, the understanding of the mechanism of resistance to TCBZ remains incomplete, with a knowledge gap in terms of its capacity to spread and strategies for control [39]. It has been suggested that resistant fasciolid strains may have alterations in drug uptake, efflux, and detoxification, including the conversion of TCBZ sulfoxide into the less active forms. However, this has not been verified in large studies using other parasite strains. Poor response to TCBZ may also be attributed to its poor water-solubility and limiting drug concentration in the organs [40–42].

In contrast to veterinary medicine where other treatment options for *Fasciola* exist, there is no documented strategy for the management of TCBZ treatment failure in humans. To minimize the development of drug resistance, the use of synergistic drug combinations has been suggested [43]. However, this approach carries the risk of building up resistance to multiple drugs [44].

Although the small sample size has limited the scope of this study, to the best of our knowledge, this is the first report of TCBZ failure in humans with acute fascioliasis in Egypt. Further multicenter randomized studies including larger sample sizes are required to evaluate predictors of TCBZ failure. This will help to determine the optimum timing for repeating TCBZ after failure of the initial dose. Also, further research is urgently needed to find new therapeutic alternatives to TCBZ for controlling fascioliasis.

In this first report of TCBZ failure in acute human fascioliasis in Upper Egypt, NTZ proved to be partially effective.

## Supporting information

S1 Checklist STROBE checklist. (DOCX)
