# Correction: Evaluation of nitazoxanide treatment following triclabendazole failure in an outbreak of human fascioliasis in Upper Egypt

**DOI:** 10.1371/journal.pntd.0008443

**Published:** 2020-06-17

**Authors:** Haidi Karam-Allah Ramadan, Waleed Attia Hassan, Nahed Ahmed Elossily, Alzahraa Abdelraouf Ahmad, Adnan Ahmed Mohamed, Alaa Soliman Abd- Elkader, Eman M. Nagiub Abdelsalam, Hani M. J. Khojah

In the methodology section of the abstract the term “pathological” was used incorrectly and should be “biochemical” instead. The corrected sentence reads: “The remaining cases received additional doses of TCBZ with complete clinical, biochemical, and radiological resolution.”

There is a typographical error in the footnote of Table 5. In the updated Table 5, the reference range for platelet count in the footnote has been updated to 150,000–450,000/µL.

**Table 5:** Laboratory data of the studied patients who received first line triclabendazole.

	Non- responders to TCBZ (n = 30)	Responders to TCBZ (n = 37)	*P* value
	Mean ± SD	Mean ± SD	
**Hb level** (g/dl):	11.80 ± 1.13	11.81 ± 0.90	0.930
**WBCs** (cells/µL):	17.46 ± 12.25	10.27 ± 3.92	0.008^*^
**Eosinophils % before treatment**	26.72 ± 13.21 *Range = 14–40%*	30.47 ± 15.18 *Range = 7–70%*	0.081
**Eosinophils % after treatment**	20.00 ± 11.28 *Range = 3–59%*	3.6 ± 1.7 *Range = 1–15%*	0.0001^*^
**PLT** (cells/µL)	311.60 ± 85.07	308.11 ± 75.79	0.796
**Total bilirubin** (mg/dl)	1.04 ± 0.15	1.06 ± 0.17	0.613
**ALT** (IU/L)	61.43 ± 43.44	39.62 ± 18.52	0.026^*^
**AST** (IU/L)	53.83 ± 36.05	35.78 ± 15.84	0.047^*^
**Positive stool analysis**	2 (6.7%)	5 (13.5%)	0.447
**Serology titer**	885.33 ± 391.32	977.30 ± 384.19	0.338

**Hb,** hemoglobin; **WBCs,** white blood cells; **PLT,** platelets count; **ALT,** alanine aminotransferase; **AST**, aspartate aminotransferase.

Reference ranges: Hb (male) = 14–17 g/dL; Hb (female) = 12–16 g/dL; WBCs = 4000–10,000/μL; eosinophils = 0–5%; PLT count = 150,000–450,000/μL; total bilirubin = 0.3–1.2 mg/dL; ALT = 0–35 IU/L; AST = 0–35 IU/L. Serology titer ≥ 1/320 is considered positive.

* Significant *p*-value by Fisher exact test.
