# Supplementary material for: Evaluation of nitazoxanide treatment following triclabendazole failure in an outbreak of human fascioliasis in Upper Egypt
Source: PLoS Negl Trop Dis. 2019 Sep 25;13(9):e0007779. doi: 10.1371/journal.pntd.0007779 (PMC6779272; doi:10.1371/journal.pntd.0007779)
Supplement: S1 Checklist — (DOCX) [file pntd.0007779.s001.docx]

STROBE Statement—Checklist of items that should be included in reports of ***cohort studies***

|  | **Item No** | **Recommendation** |
| --- | --- | --- |
| **Title and abstract** | 1 | Indicate the study’s design with a commonly used term in the title or the abstract  *The abstract describes the study design as “a prospective study”* |
|  |  | (*b*) Provide in the abstract an informative and balanced summary of what was done and what was found  *The abstract describes the methods and findings* |
| **Introduction** | | |
| Background/rationale | 2 | Explain the scientific background and rationale for the investigation being reported  *The background and rationale are described in the Introduction, paragraphs 1 -5* |
| Objectives | 3 | State specific objectives, including any prespecified hypotheses  *The specific aims and hypothesis of the study are stated in the Introduction, paragraphs 6 & 7* |
| **Methods** | | |
| Study design | 4 | Present key elements of study design early in the paper  *The study design is discussed in paragraphs 1-9 of the Methods section* |
| Setting | 5 | Describe the setting, locations, and relevant dates, including periods of recruitment, exposure, follow-up, and data collection  *The institutional setting is described in paragraph 2 of the Methods section; Study locations are described in paragraph 3 of the Methods section; and study timing is discussed in paragraphs 1and 6-7 of the Methods section.* |
| Participants | 6 | Give the eligibility criteria, and the sources and methods of selection of participants. Describe methods of follow-up  *Selection of the participants is discussed in paragraphs 3 & 4 & 9 of the Methods section. Methods of follow up are described in paragraph 5-8 of the Methods section.* |
| Variables | 7 | Clearly define all outcomes, exposures, predictors, potential confounders, and effect modifiers. Give diagnostic criteria, if applicable  *Outcomes are discussed in the Outcomes subsection (paragraphs 7 and 8 of the Methods section). The primary outcome of the study is whether patients were “TCBZ Responders” defined according to the WHO criteria of good Triclabendazole treatment response or “TCBZ non- Responders “for a trial with nitazoxanide and further follow up by CBC and US.* |
| Data sources/ measurement | 8* | For each variable of interest, give sources of data and details of methods of assessment (measurement). Describe comparability of assessment methods if there is more than one group  *Measurement of the outcomes for either TCBZ Responders group and non -Responders group are discussed in the Outcomes subsection (paragraphs 7 and 8 of the Methods section)* |
| Bias | 9 | Describe any efforts to address potential sources of bias  *The avoidance of common sources of bias inherent in other clinical studies, are discussed in paragraph 6 &7 of the Methods section and paragraph 2 & 4 of the Discussion section* |
| Study size | 10 | Explain how the study size was arrived at  *Sample determination is discussed in paragraph 3 of the Methods section* |
| Quantitative variables | 11 | Explain how quantitative variables were handled in the analyses. If applicable, describe which groupings were chosen and why  *Use of variables is discussed in the Statistical Analysis subsection* |
| Statistical methods | 12 | Describe all statistical methods, including those used to control for confounding  *Statistical methods are discussed in the Statistical Analysis subsection* |
|  |  | (*b*) Describe any methods used to examine subgroups and interactions  *Described in paragraph 1 of the Statistical Analysis subsection. Results are analysed by facility level.* |
|  |  | (*c*) Explain how missing data were addressed  *Missing observations are discussed in paragraph 1 of the Results and shown in the STROBE Flowchart. 7 patients were excluded as they presented with obstructive jaundice and a dilated CBD on ultrasound (suggesting chronic fascioliasis). These 7 patients underwent endoscopic sphincterotomy and extraction of the adult worm by ERCP followed by TCBZ therapy. No individual variables are missing for patients included in the analysis* |
|  |  | (*d*) If applicable, explain how loss to follow-up was addressed  None |
|  |  | (e) Describe any sensitivity analyses  *The sensitivity analysis of results was discussed in the Statistical Analysis subsection and shown in Tables 2-4, imputing the significant P value of the qualitative variables in the study* |
| **Results** | | |
| Participants | 13* | (a) Report numbers of individuals at each stage of study—eg numbers potentially eligible, examined for eligibility, confirmed eligible, included in the study, completing follow-up, and analysed  *Data collection completion rates are discussed in paragraphs 1& 2 of the Results Section. The number of patients at each group completing each phase of data collection is also shown in Fig.2* |
|  |  | (b) Give reasons for non-participation at each stage  *None* |
|  |  | (c) Consider use of a flow diagram  *Fig.2* |
| Descriptive data | 14* | (a) Give characteristics of study participants (eg demographic, clinical, social) and information on exposures and potential confounders  *Patient characteristics are presented in Table 1* |
|  |  | (b) Indicate number of participants with missing data for each variable of interest  *Presented in S2 Fig (STROBE Flowchart). There is no data missing from any patient during follow up.* |
|  |  | (c) Summarise follow-up time (eg, average and total amount)  *The follow up of patients were done as monthly visit depending on the response to treatment and as reported throughout the Results Section.* |
| Outcome data | 15* | Report numbers of outcome events or summary measures over time  *Both numbers and percentages/proportions are reported throughout the Results Section* |
| Main results | 16 | (*a*) Give unadjusted estimates and, if applicable, confounder-adjusted estimates and their precision (eg, 95% confidence interval). Make clear which confounders were adjusted for and why they were included  *Not applicable* |
|  |  | (*b*) Report category boundaries when continuous variables were categorized  *the category boundaries of continuous variables are reported in tables 1, 2 and 3* |
|  |  | (*c*) If relevant, consider translating estimates of relative risk into absolute risk for a meaningful time period  *Not relevant* |
| Other analyses | 17 | Report other analyses done—eg analyses of subgroups and interactions, and sensitivity analyses  *Other analysis was described in statistical analysis subsection* |
| **Discussion** | | |
| Key results | 18 | Summarise key results with reference to study objectives  *Results are summarized in paragraphs 8,10,12 of the Discussion section.* |
| Limitations | 19 | Discuss limitations of the study, taking into account sources of potential bias or imprecision. Discuss both direction and magnitude of any potential bias  *Limitations are discussed in paragraph 4 and the pre-final paragraph 19 of the Discussion section (in limitation subsection)* |
| Interpretation | 20 | Give a cautious overall interpretation of results considering objectives, limitations, multiplicity of analyses, results from similar studies, and other relevant evidence  *Clearly discussed in the Discussion section* |
| Generalisability | 21 | Discuss the generalisability (external validity) of the study results  *The representativeness of the sample is discussed in the paragraph of the limitations subsection.* |
| **Other information** | | |
| Funding | 22 | Give the source of funding and the role of the funders for the present study and, if applicable, for the original study on which the present article is based  *Triclabendazole treatment regimens (Egaten®, Novartis Pharma AG) were provided at the Ministry of Health and Population in a joint venture with the WHO otherwise no institutional fund was provided.* |

*Give information separately for exposed and unexposed groups.

**Note:** An Explanation and Elaboration article discusses each checklist item and gives methodological background and published examples of transparent reporting. The STROBE checklist is best used in conjunction with this article (freely available on the Web sites of PLoS Medicine at http://www.plosmedicine.org/, Annals of Internal Medicine at http://www.annals.org/, and Epidemiology at http://www.epidem.com/). Information on the STROBE Initiative is available at http://www.strobe-statement.org.
